# Quantitative criteria for choosing targets and indicators for sustainable use of ecosystems

**DOI:** 10.1016/j.ecolind.2016.08.005

**Published:** 2017-01

**Authors:** Axel G. Rossberg, Laura Uusitalo, Torsten Berg, Anastasija Zaiko, Anne Chenuil, María C. Uyarra, Angel Borja, Christopher P. Lynam

**Affiliations:** a Centre for Environment, Fisheries and Aquaculture Science (Cefas), Pakefield Road, Lowestoft NR33 0HT, UK and School of Biological and Chemical Sciences, Queen Mary University of London, 327 Mile End Rd, London E1, UK; b Marine Research Centre, Finnish Environment Institute (SYKE). Mechelininkatu 34a, P.O. Box 140, FI-00251 Helsinki, Finland; c MariLim Aquatic Research GmbH, Heinrich-Wöhlk-Straße 14, 24232 Schönkirchen, Germany; d Marine Science and Technology Center, Klaipeda University, H. Manto 84, LT 92294, Klaipeda, Lithuania; e Aix-Marseille Univ, Univ Avignon, CNRS, IRD, IMBE, Marseille, France; f AZTI-Tecnalia, Herrera Kaia, Portualdea s/n, 20100 Pasaia, Spain; g Centre for Environment, Fisheries and Aquaculture Science (Cefas), Pakefield Road, Lowestoft NR33 0HT, UK

**Keywords:** Good environmental status, Marine Strategy Framework Directive, Sustainable use, Assessment, Ecological indicators

## Abstract

Wide-ranging, indicator-based assessments of large, complex ecosystems are playing an increasing role in guiding environmental policy and management. An example is the EU’s Marine Strategy Framework Directive, which requires Member States to take measures to reach “good environmental status” (GES) in European marine waters. However, formulation of indicator targets consistent with the Directive’s high-level policy goal of sustainable use has proven challenging. We develop a specific, quantitative interpretation of the concepts of GES and sustainable use in terms of indicators and associated targets, by sharply distinguishing between current uses to satisfy current societal needs and preferences, and unknown future uses. We argue that consistent targets to safeguard future uses derive from a requirement that any environmental state indicator should recover within a defined time (e.g. 30 years) to its pressure-free range of variation when all pressures are hypothetically removed. Within these constraints, specific targets for current uses should be set. Routes to implementation of this proposal for indicators of fish-community size structure, population size of selected species, eutrophication, impacts of non-indigenous species, and genetic diversity are discussed. Important policy implications are that (a) indicator target ranges, which may be wider than natural ranges, systematically and rationally derive from our proposal; (b) because relevant state indicators tend to respond slowly, corresponding pressures should also be monitored and assessed; (c) support of current uses and safeguarding of future uses are distinct management goals, they require different types of targets, decision processes, and management philosophies.

## Highlights

• A simple quantitative method for choosing ecological indicators and target ranges is proposed.
• Sustainable use of ecosystems requires freedom of usage choice for each generation.
• Sustainability so limits any state indicator to the range from which timely recovery is feasible.
• Relevant state indicators are those that anthropogenic pressure might drive out of this range.
• The method extends to pressure- and auxiliary indicators, and suites of indicators.

## Graphical abstract

**Figure:**
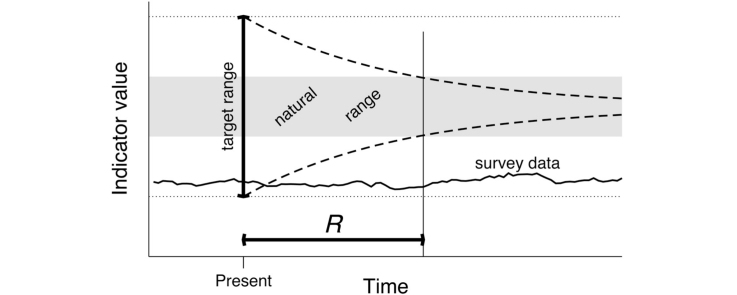

## 1 Introduction

### 1.1 From qualitative to quantitative criteria for indicator selection

Ecological indicators are increasingly being used in rule-based management schemes where indicator values outside their respective target ranges trigger management action. The question which properties ecological indicators should have for this purpose has often been addressed in the literature (Elliott, 2011 Queirós et al., 2016, Rice and Rochet, 2005). An example relevant for assessment and management of marine ecosystems is the set of criteria proposed by ICES (2001), which forms the basis of the Rice and Rochet (2005) criteria. These relate to concreteness, theoretical basis, public awareness, cost, measurability, representation through historic data, sensitivity, responsiveness, and specificity of indicators. A list by Elliott (2011) containing 18 criteria goes beyond the Rice and Rochet (2005) list, in requiring that indicators (and monitoring parameters) should be anticipatory, broadly applicable and integrative over space and time, interpretable, have low redundancy, be non-destructive, time-bounded and timely. For a detailed review and analysis of indicator selection criteria, see Queirós et al. (2016).

However, practically all published specifications of desiderata for ecological indicators and their management targets remain at a qualitative level, despite containing some quantitative components (e.g. reasonable cost in comparison with expected benefits). This has the advantage of flexibility to accommodate variation in preferences and priorities of different stakeholder groups—after all, policies manage human activities rather than the marine environment (Elliott, 2013). However, experts can vary widely in their findings when evaluating indicators according to the same criteria (Rice and Rochet, 2005), which questions the idea that such criteria provide an objective basis for indicator selection. Another disadvantage is that the scientific problem of developing indicators and monitoring programs and the scientific and societal challenge of finding appropriate target ranges for these indicators remain vaguely specified. This may lead to inconsistencies in specified target ranges, inefficient use of limited monitoring capacity, and uncertainty about the most appropriate use of research capacity for refining indicators and targets or filling potential gaps in indicator suites (Borja et al., 2012).

Ideally, a quantitative, generic, and broadly accepted framework was available for choosing indicators and setting targets, so making this a research and development task to deliver a product according to specifications, rather than a social process of finding common positions in an uncertain space. Such a quantitative framework does currently not exist. Environmental policy documents tend to specify their overall high-level objectives in a qualitative language. The purpose of this contribution is to propose, as a way forward, a quantitative interpretation of this qualitative language, which can then be tested for political acceptance. Being deliberately constructed building on just a few generic principles, our proposal is necessarily somewhat abstract and rigid, and so should not be misunderstood as a direct prescription of policy. More plausibly, it will serve as a scientifically anchored orientation point for political decision making.

As a specific policy document which is currently widely discussed in Europe, we chose to focus here on the Marine Strategy Framework Directive (MSFD; EC, 2008) of the European Union (EU). The principles being invoked for setting targets are not consistent within the community implementing the MSFD. For Cochrane et al. (2010), for example, the target is an ecosystem nearly unperturbed by humans, ICES (2014a) primarily require that ecosystem functions are not degraded, Rogers et al. (2010) and ICES (2014b) refer to abundances that can recover from perturbation or have been observed to be historically stable, and Piet et al. (2010) interpret the “safe biological limits” of fish stocks as those producing maximum sustainable yield. We shall here concentrate on policy needs under the MSFD. However, the framework we proposed might be generally useful for linking assessments of aquatic or terrestrial ecosystems to high-level policy goals.

### 1.2 The concept of sustainable use

The MSFD requires from EU member states to determine, in a collaborative manner, specific environmental targets and corresponding quantitative indicators that together represent “good environmental status” (GES). It defines GES as:

the environmental status of marine waters where these provide ecologically diverse and dynamic oceans and seas which are clean, healthy and productive within their intrinsic conditions, and the use of the marine environment is at a level that is sustainable, thus safeguarding the potential for uses and activities by current and future generations […].

The last passage is a variation of the definition of sustainable development from the Brundtland Report (World Commission on Environment and Development, 1987):

Sustainable development is development that meets the needs of the present without compromising the ability of future generations to meet their own needs.

Important is that this definition recognizes that needs of future generations might be different from current needs. By referring to “the potential for uses and activities by […] future generations”, the MSFD follows this tradition. Uncertainty about future uses, and so values, of resources naturally leads to strong notions of sustainability^1^ that aim at independent maintenance or enhancement of various forms of natural and non-natural capital (Figge, 2005). Contrastingly, weak sustainability permits substitution of natural with manufactured capital, implicitly assuming good knowledge of their respective future values (Figge, 2005). Correspondingly, we say here “*strongly sustainable”* for *use* of the environment that does not constrain usage choices and capabilities of future generations, and “*weakly sustainable”* for *use* that simply can be continued indefinitely in its current form (conceivable are even weaker notions). The distinction between the two concepts is briefly summarized in Table 1.

The best-known example of usage of “sustainable” in our weak sense in the marine ecology context is “maximum sustainable yield” (MSY). Management for MSY alone does not necessarily imply sustainability by the stronger definition, because changes to the wider ecosystem resulting from exploitation may be irreversible. The MSFD refers to weakly sustainable use, for example through the adjective “productive” in the GES definition above and in a clarifying Commission Decision (EC, 2010), which explicitly specifies exploitation at MSY as a target.

Our considerations here concentrate on strongly sustainable use, thus marking the limits within which weakly sustainable use options can be explored. From above considerations it follows that constraints imposed by strong sustainability will generally be weaker than those following from specific weakly sustainable use objectives; a potential source of confusion to keep in mind.

The operationalization of the strong concept of sustainable use in the context of marine management has been subject of extensive discussion in the work of the International Council for the Exploration of the Seas (ICES, 2005, ICES, 2010, ICES, 2013). ICES argued that, since the needs and preferences of future generations are unknown to us, sustainable use means not to perturb the ecosystem to such a degree that recovery from these perturbations is impossible or unacceptably slow (see also FAO, 2009). In other words, under sustainable use the system must remain capable of recovering to an unperturbed state over an acceptable time span.

When making this idea operational, two points need bearing in mind. Firstly, since the management objective is sustainable use in the present rather than in the past, the unperturbed state is not necessarily a historic or pre-historic state, but the state that would be reached in the long term if all anthropogenic pressures were removed. Secondly, the unperturbed state itself is not fixed but undergoes natural fluctuations.

Developing a quantitative interpretation of sustainable use, ICES (2010) proposed to focus indicator selection on ecosystem components that (1) are under pressure and (2) for which recovery from pressures is slow or impossible. Indicators are then chosen to quantify the state of these components or features, called “vulnerable components” below, and the pressures on them. This method, however, leaves open the problem of deriving target values for these indicators.

Here the approach of ICES is therefore reversed. A rule is proposed for setting target ranges for arbitrary quantitative indicators of ecosystem state such that, for ecosystem components that are not vulnerable in the sense above, the targets will “automatically” be met under almost all circumstances, while indicators relating to vulnerable components are easily driven out of their target ranges under inappropriate management, which is then interpreted as unsustainable use. That is, the rule for setting target ranges implicitly selects indicators critical for monitoring sustainable use, and these implicitly identify vulnerable ecosystem components, so focusing assessments and management on protecting the latter.

The selected state indicators are complemented with a set of corresponding pressure indicators, and potentially with additional indicators quantifying state along causal chains linking anthropogenic pressures to vulnerable ecosystem components.

## 2 The proposal

### 2.1 Choosing target ranges for state indicators

The rule for choosing indicator target ranges proposed here contains a single free parameter, the longest acceptable mean recovery time R (precisely: the largest acceptable expectation value of time to recovery). The value of R is a matter of societal choice. It could be related, e.g., to the duration of policy cycles or the human life cycle. According to a definition by the FAO (2009), for example, ‘significant adverse impacts’ on ecosystems will typically have recovery times exceeding 5–20 years. Consistent use of the same value of R when setting target ranges for different indicators improves consistency among management goals. Society might require comparisons of the implications of different choices of R in order to make an informed decision on its numerical value. We propose that, to remain consistent with intergenerational freedom of choice, R should not exceed the approximate human generation time of 30 years, and assume R≈30years in examples we discuss.

Now, let I stand for any univariate indicator of ecosystem state. The indicator is here understood as being defined directly in terms of ecosystem state variables, rather than by a protocol to measure these. Without anthropogenic pressures, the value of I would relax to and then naturally fluctuate around some typical value. The resulting distribution of values I can be called its pressure-free, and, in this sense, *natural distribution*.

**Fig. 1:**
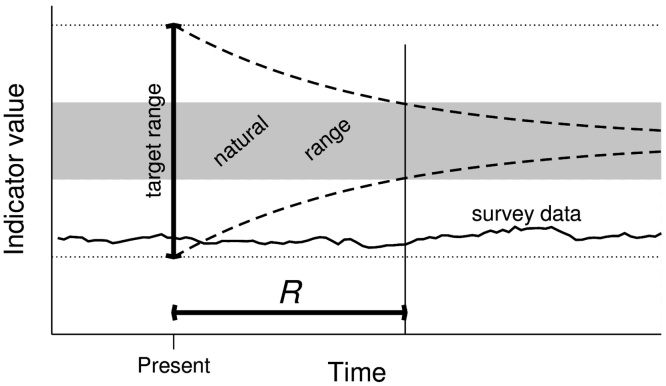
Illustration of proposed approach for choosing target ranges. The target range of an indicator is determined as the range of values from which it takes, on average, at most a time R to reach the natural range in a hypothetical situation without anthropogenic pressures. Dotted lines indicate the width of the target range, dashed lines hypothetical average relaxation trajectories, the grey area the natural range, and the ragged solid line a conceivable trajectory of the indicator for an ecosystem in strongly sustainable use. In practice, the target range may need to be narrowed to take measurement uncertainty and model uncertainty into account.

One can define a natural range of variation $\left[I_{\text{low}},I_{\text{high}}\right]$, for example by chosing $I_{\text{low}}$ as the 2.5% quantile of the natural distribution, and $I_{\text{high}}$ as the 97.5% quantile. Under natural conditions, the indicator is then in the natural range 95% of all times. Because direct observation data corresponding to natural or pristine conditions does not necessarily exist, inferential methods to determine natural ranges will often be required. We now propose to choose the target range for any indicator as the range of values from where the mean time to reach the natural range when all pressures are, hypothetically, removed is not larger than the acceptable mean recovery time R. The idea is illustrated in Fig. 1. Management under this rule implies that, after an average transition period R, future generations can use the corresponding ecosystem component in any form that would have been almost certainly possible under natural conditions, provided “almost certain” is interpreted as meaning 95% probability.

The indicator’s natural range depends on external factors, in the case of the MSFD described as “the associated physiographic, geographic, geological and climatic factors”. Complicating, Earth’s climate is on a trajectory of directed long-term change, and the natural range corresponding to current climatic conditions gradually changes. Target ranges should be chosen such that relaxation to the natural range within R on average is possible even though it changes over time.

### 2.2 Choosing relevant state indicators

By our proposal, all aspects of ecosystem state are potentially relevant. These including, e.g., the physical seascape, water temperature and flows, chemical water composition, the structuring elements of the ecosystem such as habitat-forming species, top predators, and key resource species, but also endangered species, groups or habitats, and high-level properties such as species richness, community biomass and production. If follows from our rule of choosing target ranges that among these the state indicators that are relevant in practice (below “relevant indicators”) are those which are outside their target ranges or likely to be pushed out of their target ranges by prevalent or foreseeable anthropogenic pressures. Sets of candidate state indicators can initially be scanned for relevance by asking if their recovery to the natural range can conceivably last longer than R.

### 2.3 Choosing relevant pressure indicators and their targets

We propose to choose the combined target ranges of pressure indicators in such a way that, when pressures are maintained indefinitely within target ranges, all ecosystem state indicators return to their target ranges and then remain within these ranges during 95% of time.^2^ To cope with empirical uncertainty over pressure-state relationships, an adaptive management scheme where pressure target ranges are iteratively revised based on observed changes in state will often be adequate. Analogously to the state indicators, relevant pressure indicators are those which are outside or likely to be brought outside their target ranges, and they can be found by a similar scanning procedure.

### 2.4 Causal relations and supporting indicators

Some vulnerable ecosystem components are not or not only affected by direct anthropogenic pressures, but also indirectly *via* causal chains through other ecosystem components (Borja et al., 2010b). A well-known example are changes in populations at higher trophic levels caused, through bottom-up control, by populations at lower trophic levels, in turn influenced, e.g. by fluvial nutrient input. If pressure-state relationships along these causal chains are not well understood, monitoring of intermediate ecosystem components, e.g. abundance of primary or secondary producers, can play an important role in supporting decision making by managers. Existence of causal “webs” rather than linear chains heightens this need (Borja et al., 2010b). Effective supporting indicators will have comparatively well-understood causal links to both anthropogenic pressures and vulnerable ecosystem components, so maximising the information on causal relations between pressures and states. Target ranges for such supporting state indicators can be determined following the same logic as those for direct anthropogenic pressures.

### 2.5 Suites of indicators and correlations between indicators

To adequately capture the status of complex marine ecosystems, large sets of indicators are often proposed. The question then arises by which criterion potentially redundant indicators could be identified and eliminated. Within the present framework, a natural answer arises as follows: consider a situation where, under current and foreseeable pressures, some formula predicts the values of one indicator I in a suite of state indicators from those of the other indicators up to a differenceD. Then I can be replaced by D in this suite without loss of information. When D is not a relevant indicator by our proposal, D (and I) can be removed from the suite.

Situations can also arise where relevant state indicators are ecologically coupled so that the mean relaxation time of one indicator depends on the values of other indicators, but the coupling is not strong enough to justify disregarding any of them by the logic above. We suggest two ways of dealing with this situation: (1) to set the target ranges of such indicators depending on the current values of other indicators, or (2) to find target ranges for all indicators such that, as long as all are within target ranges, each will relax to its natural range within R, no matter what the values of the others. Both options reduce to our original proposal if indicators are uncoupled. Option 2 might lead to narrower target ranges, but is more easily administered.

### 2.6 Precautionary buffers

A precautionary approach to management can be implemented following logic very similar to that applied in traditional fisheries management (ICES, 1998): after determining the target range for an indicator, it is narrowed down to take measurement errors in determining its value and model uncertainties in the determination of the target range into account. Model uncertainties can affect determination of both natural range and mean recovery time.

When quantitative estimates of measurement and model uncertainty are available, the precautionary target range could be chosen so that (1) mean recovery time remains ≤R also when taking both kinds of uncertainty into account and (2) the correct indicator value will be within the correct target range in, say, at least 95% of cases. Depending on the circumstances, one or the other condition will be stronger.^3^ Management aiming to respect target ranges of several indicators, while taking uncertainty in system dynamics into account, could make use of the viability kernel method (Cury et al., 2005, Mullon et al., 2004), which works independent of the criteria by which target ranges are defined.

For pressure indicators, not only uncertainty in target ranges of subsequently affected state indicators needs to be considered, but also in the pressure-state relationships and the actual magnitude of pressures.

It is an economic decision to balance the costs of monitoring and research to improve knowledge of pressure-state relations with the opportunity costs of wider precautionary buffers when uncertainties are high.

### 2.7 Is our science ready?

The importance of recovery times for the management of marine resources has long been recognised in the literature (Borja et al., 2010a, Duarte et al., 2013, Verdonschot et al., 2012). The quantitative application of this concept for indicator selection proposed here is just the logical extension of this line of thought, and can build on rich previous research determining recovery times and modelling recovery processes.

**Fig. 2:**
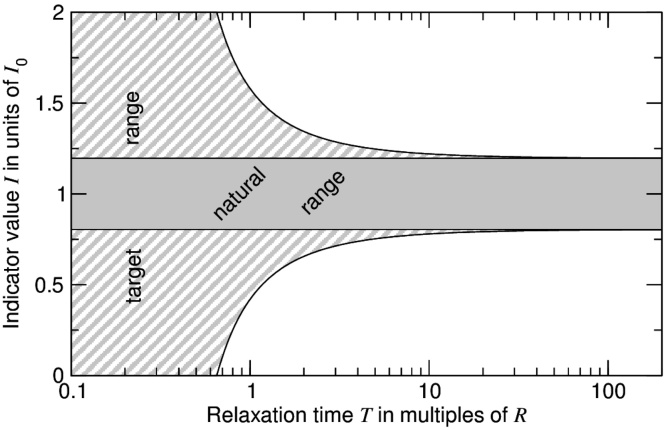
Dependence of target range for strongly sustainable use (hatched & grey area) on indicator relaxation time T for the linear model Eq. (1). The natural range (grey area) is shown for comparison. Calculation assumes a coefficient of variation for the natural distribution of 0.1.

The demands of our proposal on the accuracy at which recovery times can be determined might be comparatively low. As shown in Appendix (Fig. 2), rather coarse estimates will often be sufficient, either because recovery is fast compared to R and so the target range too wide to be relevant, or because recovery is so slow that little variation beyond the natural range is tolerated.

## 3 Examples

Next we apply our criteria to several types of candidate indicators to explore feasibility and likely practical implications of our proposal. In each case we estimate the magnitude of relaxation times and/or the approximate widths of target ranges, and, based on this, identify the candidates relevant for strongly sustainable use. While the focus is on indicators likely to pass this test, not all candidates we consider do. Overall, we find that sufficient ecological understanding is available to carry out the proposal, and that computation of reliable target ranges would be possible with moderate extra effort.

### 3.1 The Large Fish Indicator

The Large Fish Indicator (LFI) is defined as the proportion by biomass of fish caught in a given survey that are longer than a defined length threshold. For the North Sea demersal fish community, sampled by the International Bottom Trawl Survey in quarter 1, the agreed length threshold is 40 cm (Greenstreet et al., 2011). A target range LFI ≥ 0.3 has previously been set on the basis of pre-1980 data and the view that the early 1980s were “the last period when science experts considered fishing to be generally [weakly] sustainable in the North Sea” (Greenstreet et al., 2011). Because recovery of fish community size structure has been shown to be slow (Fung et al., 2013, Rossberg, 2012, Shephard et al., 2013, Shephard et al., 2012), it is desirable to identify a target range consistent with strong sustainability.

The natural range of variability of the LFI is not known, but simulation studies (Fung et al., 2013, ICES, 2011) predict that indicator values of 0.5 or more could be reached if pressures where lower. Without any fishing, simulations by Fung et al. (2013, Fig. S5a) predict indicator values close to 0.8. Assuming a coefficient of variation for LFI of 0.05 in its natural distribution, so that the 2.5% quantile corresponds to about 90% of the mean undisturbed value, simulations by Fung et al. (2013, Fig. 7) predict that recovery from LFI ≈ 0.5 would take around 30 years and recovery from LFI ≈ 0.25 around 35–40 years. This suggests that LFI ≥ 0.3 is a reasonable target range if R is on the order of 30 years.

Besides being a state indicator for a vulnerable ecosystem component (fish community size structure), the LFI also signals pressures on marine biodiversity. Specifically, prolonged unselective fishing at a rate such that LFI remains near 0.25 leads to extirpation of nearly a third of all large fish species in simulationsFung et al. (2013, Fig. 6a). These extirpations could represent declines of vulnerable components of local biodiversity, even when they do not impede recovery of LFI itself.

### 3.2 Indicator species

#### 3.2.1 General considerations

The use of population sizes (or the correlated spatial extent) of selected “indicator species” as indicators for community or environmental status has drawn scepticism from both ecologists (Lindenmayer and Likens, 2010) and jurists (Kelly and Caldwell, 2013). Our proposal supports this scepticism: population sizes of species in communities tend to fluctuate, and exhibit little tendency, if at all, to revert to a preferred value (Kalyuzhny et al., 2014, Korhonen et al., 2010). On longer time scales this leads to the well-documented species turnover (Magnuson et al., 1994). The natural range of variation of species population sizes thus extends from fairly large values (Sec. 14.6) down to effectively zero. Corresponding indicators would not be relevant in the sense used here. This does, however, not preclude the relevance of community-level indicators derived from population sizes or presence/absence of member species (Faith and Pollock, 2014). In fact, alpha diversity is known to be sensitive to pressures but in unperturbed communities remarkably stable through time (Vellend et al., 2013), as theoretically expected from a control of alpha diversity through structural stability constraints (Rossberg, 2013).

Population size or extent of an individual species can potentially be a relevant indicator when this species is under a particular, manageable pressure, when the species is vulnerable to global or regional extinction (from which recovery would be slow or impossible), or when the set of its actual or possible competitors is so small that natural species turnover cannot unfold. For top predators, all three of these criteria are likely to be satisfied, which justifies the use of species-level indicators in this case, as illustrated by the next example.

#### 3.2.2 Abundance of seals as an indicator

Bounty hunting, encouraged in order to decrease the mortality of fish, caused the collapse of the Baltic grey seal (*Halichoerus grypus*) population from approximately 80,000–100,000 individuals in the early 1900s to ca. 20,000 individuals in 1940s (Elmgren, 2001, Harding and Härkönen, 1999). Ceased hunting did not result in recovery of the population, however. Most probably due to environmental pollution harming reproduction, the population further decreased to approximately 2000 in the late 1970s (Boedeker et al., 2002, Harding and Härkönen, 1999). As these pressures have been relieved or removed since the early 1990s, the population has increased to ca. 28,000 individuals today (Harding et al., 2007, Harding and Härkönen, 1999, Härkönen et al., 2013). The population growth rate has been >10% yearly between the early 1990s and mid-2000s, but slowed down to about 6% in the 2010s (Härkönen et al., 2013).

The Baltic Marine Environment Protection Commission (HELCOM) monitors the seal population size and growth rate through a core indicator (Härkönen et al., 2013). A target has been set for the population growth rate to ≥10% yearly, but none for population size. In addition to hunting and environmental pollution, anthropogenic threats to seals include drowning in fishing gear, and decrease in food quality and spread of parasites due to changes in the food web. A population size of 80,000–100,000 (Harding and Härkönen, 1999) can be used as an estimate of the natural range for the Baltic Sea grey seal. Assuming a constant 10% yearly population growth rate, a population size of 5050 individuals would be enough to rebuild the population to $N_{\text{low}}$ = 80,000 individuals in R = 30 years, and r= 6% yearly growth would require 15,800 individuals or more. Assuming, more realistically, logistic growth with a carrying capacity of K=100,000 individuals, one obtains a lower limit of the strongly sustainable population target range of $N_{\text{lim}}=K{\left[1+e^{rR}\left(K/N_{\text{low}}-1\right)\right]}^{-1}=40,000$ individuals. More detailed models might take dependencies, e.g. on food availability, into account as explained in Section 2.5.

As the seal population has increased, predation on valuable fish and damages caused by seals to fishing gear are increasingly seen as problems (Holma et al., 2014, Varjopuro, 2011). On the other hand, it has been proposed that abundant seal populations could boost tourism in coastal communities. Finding a balance between competing services and uses of the marine ecosystem has been recognized as a challenge to be solved (e.g. the ECOSEAL project, http://www.ecosealproject.eu/). By our proposal, the ultimately targeted size of the Baltic grey seal population should not lie below $N_{\text{lim}}$ to be consistent with strong sustainability.

### 3.3 Secchi depth

Eutrophication is one of the major pressures at sea, where it affects several other ecosystem components: the food web, sea-floor integrity, and biodiversity (Cloern, 2001). Increases of phytoplankton biomass are primarily caused by anthropogenic nutrient enrichment in the water. One of the key aims of the Baltic Sea Action Plan is a “Baltic Sea unaffected by eutrophication”, and two indicators related to this aim are water clarity (Secchi depth) and chlorophyll a concentration, which are used as proxies for phytoplankton abundance.

Secchi depth measurements from 1900 to 1920 in the northern Baltic Sea range between 5 and 15 m, with mean values around 9 m (Fleming-Lehtinen and Laamanen, 2012). This can be considered the natural range, as anthropogenic nutrient loading was low at that time. Secchi depth in these basins has since decreased, reaching 2–9 m during the last decade (Fleming-Lehtinen and Laamanen, 2012). This change is concurrent with increases in nutrient loading and nutrient concentrations in the water. HELCOM targets for Secchi depth in the various basins of the Baltic Sea range between 5.5–8.5 m (Fleming-Lehtinen et al., 2014). These targets are set based on the principle of allowing 25% deviation from the undisturbed state.

While anthropogenic nutrient enrichment is the major driver for nutrient concentrations in the water, eutrophication abatement is complicated by internal loading, a process that recycles sedimented nutrients back to the water column (Pitkänen et al., 2001). Internal loading forms a vicious cycle (Vahtera et al., 2007), as it increases in non-oxygenated sediments, which again increase due to increased sedimentation of phytoplankton biomass. Internal loading can delay the decline of nutrient in the water after a reduction in anthropogenic input. A similar delay must be expected for Secchi depth.

Models suggest that response times of nutrient concentrations are of the order of 40 years (Ahlvik et al., 2014, Kiirikki et al., 2006, Neumann and Schernewski, 2008). Linking these models to empirical models for Secchi depth (Savchuk and Wulff, 2007), quantitative target ranges for Secchi depth consistent with strong sustainability could be derived to inform the ongoing debate on target setting (Ahtiainen et al., 2014).

### 3.4 Genetic diversity

Operational indicators to quantify genetic diversity within populations have been defined since the 1990s (Chenuil, 2006, Petit et al., 1998). Loss of genetic diversity is of concern because of its detrimental impacts on population resilience (Frankham, 2005, Frankham et al., 2002, Schwartz et al., 2007). Genetic diversity will decline sharply during periods of small population size, and laboratory and field studies have documented negative responses to various environmental and anthropogenic pressures (Ozerov et al., 2013, Pini et al., 2011, Taris et al., 2006). Natural variability in populations and the environments can be expected to determine natural variability in genetic diversity. Recovery dynamics of local genetic diversity is understood to result from two processes, mutation and immigration, which exhibit contrasting dynamics. The rate of accumulation of mutations is proportional to the product of the mutation rate per locus per generation, the effective population size (Wright, 1938), and inverse generation time (Baer et al., 2007, Kimura, 1984). For higher organisms, e.g. vertebrates, corresponding time scales easily exceed 30 years. With regular immigration from neighboring or distant populations, recovery can be much faster. Hence, genetic diversity can be a relevant indicator for small populations of long-living species, in particular when these are relatively isolated or experience similar pressures over broad spatial scales.

### 3.5 Non-indigenous species indicators

Finally, we consider an important example for which application of the proposed framework is not obvious: choices and target ranges for pressure and state indicators related to non-indigenous species (NIS). Invasion of NIS is often irreversible and so direct recovery impossible (Thresher and Kuris, 2004). Yet, compared with natural species turnover, the fact alone that NIS invade local communities and there compete with native residents might, at regional level, not be an issue (loss of global biodiversity through homogenization of communities notwithstanding). However, invasions by NIS differ from species turnover by natural dispersion in being more likely to go through a phase of rapid population expansion with strong impacts on the ecosystem. At the climax of this expansion phase the affected ecosystem can be driven out of its natural range of variation, but these disruptions differ from case to case. Fortunately, there is mounting evidence that the expansion phase is generally followed by an adjustment phase at which the invader’s population and its impact on the ecosystem decline to less disruptive, in cases even beneficial, levels (Blackburn et al., 2011, Reise et al., 2006, Zaiko et al., 2014).

Our proposal can be adapted to the case of NIS if one assumes this boom and bust scenario to be the rule (Williamson, 1997), while disregarding cases where the long-term impacts remain high compared to those of natural turnover. One can then interpret the rate of NIS arrivals in an ecosystem as the pressure, and the aggregated disruptive impacts NIS cause before reaching their late adjustment phases as the resulting change in state. The impacts can be considered strongly sustainable if these disruptions would, without new arrivals of NIS, on average decline within time R to levels typical for natural turnover.

The quantification of the level of disruptions is complicated by the idiosyncrasy of NIS impacts. Among frameworks suggested for quantifying bioinvasion impacts (Copp et al., 2009, Molnar et al., 2008, Nentwig et al., 2010), the Biological Pollution Level (BPL) assessment method (Olenin et al., 2007) has been recommended as a robust and standardized indicator in the context of the MSFD (Olenin et al., 2010). It has been tested in assessments of the impacts of single and multiple NIS at various scales (Olenina et al., 2010, Zaiko et al., 2011). However, no unambiguous target range has been proposed for it, yet.

Recovery times in this interpretation depend on the pattern of boom and bust cycles, which may vary depending on intrinsic or extrinsic factors (Strayer and Malcom, 2006). For zebra mussels in Irish freshwater ecosystems, for example, Zaiko et al. (2014) document recovery times on the order of only ten years since arrival and five years after maximum impact.

## 4 General implications

### 4.1 Target ranges can differ from natural ranges

Target ranges for indicators are frequently chosen as the indicator values thought to represent ecosystems unperturbed or only slightly perturbed by human interference, see, e.g. the European Water Framework Directive (EC, 2000). The present proposal supports this approach for ecosystem components with relaxation rates much slower than 1/R (see Appendix, Fig. 2). For components that relax faster, the proposal leads to broader indicator target ranges and, crucially, supports this by a simple rationale.

### 4.2 Importance of pressure indicators

If an indicator has a long relaxation time, its current value can be interpreted as representing the cumulative effect of pressures over a corresponding time span (see Appendix, Observation 1). The indicator value can change rapidly only when, temporarily, pressures far exceed the level corresponding to strongly sustainable use. If pressures become weaker or are entirely removed, the impact of previous cumulative pressures initially remains and only slowly fades away as the indicator recovers. The analogy to “mining” has been invoked (Herrick et al., 2006). Effectiveness of management on shorter time scales must therefore be assessed not only directly through state indicators, but also indirectly based on corresponding pressure indicators. Hence, pressure indicators have a particularly important role to play in the present interpretation of sustainable use.

A pattern one expects to see frequently in time series of state indicators for vulnerable ecosystem components is a rapid decline in phases of unmanaged overuse, followed by slow recovery to a baseline after management became effective (Duarte et al., 2013). Recovery at the same rate as collapse can not generally be expected. Symmetric patterns of decline and recovery are more characteristic of rapidly recovering indicators or natural fluctuations under managed sustainable use.

### 4.3 Signal-to-noise ratio, monitoring intensity and costs

Relevant state indicators have narrow ranges of natural variability, and yet are responsive to lasting pressures. In the language of engineering, their signal-to-noise ratio is high.

Due to the inherently slow dynamics of relevant indicators and their high signal-to-noise ratio, monitoring intensity does not need to be high, unless there are concerns that present anthropogenic pressures lead to rapid changes in indicator values. Relevant state indicators therefore tend have comparatively low monitoring costs.

### 4.4 Exceptionality of relevant indicators

Mathematical considerations suggest that, potential state indicators with long relaxation times tend have broad natural ranges of variability (Appendix, Observation 5), because they integrate the impacts of natural fluctuations over long time. Co-occurrence of slow dynamics and small variability, as required for indicator “relevance”, implies that underlying ecosystem properties remain mostly unaffected by the inherent variability of other properties. Often this will be the case because indicator dynamics is governed by general ecological or physical principles (e.g. conservation laws) that inhibit strong fluctuations. Indicators of high relevance by the present proposal therefore can be expected to be rather uncommon among conceivable state indicators at large.

When indicator dynamics are governed by general ecological or physical principles it is often possible to approximate dynamics and responses to pressures by simple management models. These management models can inform choices of pressure indicators and their target ranges, as well as management practices to ensure sustainable use. We therefore expect that relevant indicators by the present proposal are among those for which effective management schemes can rather easily be developed.

### 4.5 The importance of specific use targets

It is desirable that, within the boundaries of strong sustainability, the marine ecosystem provides high levels of services to society. These should be used sustainably in the weak sense. The particular mix of services, however, depends on societal preferences. Some societies might have strong preferences for recreational uses; others might value decomposition of pollutants higher. Management targets for weakly sustainable uses and corresponding indicators are therefore unlikely to derive from simple general criteria. The problem is much more complex (Elliott, 2011), yet addressing it is paramount, because the management objective of strong sustainability on its own is insufficient for achieving the societal benefits it is meant to enable.

Returning to the analogy between the precautionary approach to fisheries management and our proposal here, the historic lesson must be recalled that the boundaries of strong-sustainability target ranges might effectively become management targets in the policy process, with detrimental effects for ecosystem functioning and services. It was not long after ICES (1998) established their formulation of the precautionary approach that official ICES advice warned of this issue (ICES, 2002), increasingly so since 2004:

*Risk aversion, based on the precautionary approach, defines the boundaries of management decisions for sustainable fisheries. Within these boundaries society may define objectives relating to benefits such as maximised long-term yield, economic benefits, or other ecosystem services. The achievement of such objectives may be evaluated against another set of reference points, target reference points, which may be measured in similar dimensions as limit reference points but which may also relate to money, food, employment, or other dimensions of societal objectives. […] setting targets for fisheries management involves socio-economic considerations. Therefore, ICES does not propose values for Target Reference Points […]. This means that […] exploitation of most stocks is likely to be sub-optimal, i.e. the long-term yield is lower than it could be.*

*[…] Managers are invited to develop targets and associated management strategies*

ICES (2004), original emphasis

Only recently MSY as a use objective was incorporated into the Common Fisheries Policy (EU, 2013).

### 4.6 Alignment with prevailing approaches

Comparison of the approach laid out here with commonly proposed qualitative criteria for choosing indicators (Queirós et al., 2016) shows them to be either aligned with these criteria or to be unrelated to them. An example for good alignment is the criterion of cost-efficiency, which, as explained above, is expected to be naturally satisfied by many indicators for the state of vulnerable ecosystem components. Examples for criteria that appear unrelated to the current proposal are the concreteness and the easy interpretability of the metrics used (Elliott, 2011). The unrelated criteria can be taken into account alongside those proposed here.

The only criterion for indicator selection that is frequently mentioned in the literature but perhaps incompatible with the present proposal is the responsiveness of indicators to management measures. We proposed to address this using pressure indicators and other supporting indicators.

Our proposal develops earlier suggestions for an operational definition of GES presented by Borja et al. (2013) by separating the characterizations of weakly and strongly sustainable use. Another distinction of our proposal from the current general understanding is the recognition that not all characteristics of ecosystems are naturally resilient (i.e. recover rapidly and predictably from pressures). Management should pay attention to potential further deterioration of resilience, but of primary concern should be ecosystem components for which resilience is naturally low.

## 5 Conclusions and policy implications

We proposed a systematic, quantitative approach to select indicators and their target ranges for the purpose of assessing strong sustainability of ecosystem use. The approach offers a rationale for improving consistency among targets and focusing investments into indicator research and monitoring. To close, we highlight three overarching implications of the proposal that are likely to stand out in future developments of MSFD and similar policy instruments.

Firstly, proposals for targets of MSFD indicators often still aim at restoring natural or near-natural ecosystem states. This is not always necessary when the policy goal is sustainable use. Here we provided an argument for the choice of alternative, broader target ranges.

Secondly, relevant state indicators, by our proposal, will almost always be paired with corresponding pressure indicators or sets of pressure indicators. Situations where either a state or a pressure indicator are sufficient to characterise the status of an ecosystem component are those where the relevant recovery times are comparatively small (Appendix, Observation 3), implying that these ecosystem components are likely to be resilient to pressures and therefore not of primary conservation concern (Appendix, Observation 4).

Thirdly, the setting of indicator target ranges for strongly sustainable use and of target ranges or values corresponding to particular use objectives should be clearly distinguished in the policy process. Authority for setting these types of targets might even be assigned to different bodies. An example where such a separation is *de facto* in place is EU fisheries management. The Common Fisheries Policy (EU, 2013) now regulates the setting of fishing quotas in accordance with MSY objectives, while respecting environmental constraints are defined, among others, by the MSFD. The MSFD, in turn, leaves room for pragmatic fisheries management. The two policy instruments are administered by different departments of the European Commission.

## 6 Appendix: mathematical analyses

In this Appendix a minimal mathematic model is introduced that describes relaxation of state indicators to some natural range and responsiveness of state indicators to pressures and environmental fluctuations. The model is then analyzed mathematically in order to develop an understanding of the general relationships between state indicator dynamics, their responsiveness to pressures, and the implications for indicator target ranges.

In the model, the indicator value changes because of (i) natural recovery to a value corresponding to an undisturbed state, (ii) external pressures and (iii) uncontrolled natural fluctuations. Specifically, it assumes a dependence of the value of an indicator I(t) on time t to follow

(1) $$\frac{dI\left(t\right)}{dt}=-\frac{\left[I\left(t\right)-I_{0}\right]}{T}-cP\left(t\right)+\text{noise.}$$

This model is a direct translation of our general understanding of indicator dynamics: The indicator value changes (“dI(t)/dt”) because of (“=”) natural recovery (“$-\left[I\left(t\right)-I_{0}\right]/T$”) to a value corresponding to an undisturbed state (“$I_{0}$”), because of external pressures (“P(t)”) and because of uncontrolled natural fluctuations (“noise”). It is legitimate to think of the three terms on the right hand side to be mechanically independent contributions with magnitudes controlled by independent mechanisms, so that the values of the constants T and c and the strength of the noise are independent parameters. Equations of the type above are mathematically well studied. An excellent exposition of the relevant mathematics in easily accessible form can found in the book by Gardiner (1990).

The constant c denotes the sensitivity of the indicator to the pressure P(t). The value of this constant can in principle be determined by monitoring the rate at which the indicator changes (dI(t)/dt) when suddenly a large constant pressure P(t)=P is applied. The value of c then follows as c≈−[dI(t)/dt]/P. It can be positive or negative. For simplicity, c is here assumed positive, so that the indicator declines when a pressure is applied.

The parameter T denotes the relaxation time constant of the indicator. When noise is negligible, T is the time it takes the indicator I(t) to reduce the distance to the equilibrium $I_{0}$ from its current value to 40% (=exp(−1)) of this value in absence of pressures.

The solution of Eq. (1) is

(2) $$I\left(t\right)=I_{0}+\overset{t}{\underset{-\infty }{\int }}\exp\left[-\left(t-\tau \right)/T\right]\left[-cP\left(\tau \right)+\text{noise}\left(\tau \right)\right]\text{d}\tau \text{.}$$

The deviation of I(t) from $I_{0}$ is proportional to a weighted sum over previous pressures and previous noise, with weights decaying exponentially as exp[−(t−τ)/T], where τ denotes points in time in the past (i.e. before t). This weight factor is of the order of magnitude of 1 over an approximate time span T, and then decays to smaller values.

When the “noise” is negligible and a constant pressure P(t)=P is applied over a time that is long compared to T, the indicator will eventually relax to a constant value

(3) $$I\left(t\right)=I_{\text{eq}}=I_{0}-TcP\left(t\right)\text{.}$$

When pressure changes though time but these changes are slow compared to T, this formula is still a good approximation.

**Observation 1** Eq. (3) implies that, in general, large relaxation times T imply a high sensitivity of the equilibrium value $I_{\text{eq}}$ to pressures.

**Observation 2** For pressures that change slowly compared toT, there is a direct functional relationship (here linear) between the pressure P and the state indicatorI(t).

Most kinds of pressures are not expected to remain constant or approximately constant over the timeR. With this in mind, we arrive at

**Observation 3** Direct functional relations between pressure P(t) and state indicators I(t) hold only for state indicators with relaxation times T considerably shorter thanR.

The “noise” term in Eq. (1) describes environmental effects that drive natural fluctuations in the indicator value.^4^ In the presence of noise the indicator does not reach the equilibrium value $I_{\text{eq}}$ given by Eq. (3) when the pressure is constant or absent, but fluctuates around this value. The width of the range of fluctuation (which is, for the present model, independent of pressure P) increases not only with increasing strength of the ”noise”, but, complicating, also with increasing autocorrelation in these fluctuations: the slower these fluctuations, the stronger their impact on I(t).^5^ Yet, as a general rule, it follows, by Eq. (2), from the additivity of the effects of noise on I(t) over a recent time interval of approximate durationT, and the randomness of the noise (by definition), that the mean *squared* deviation of I(t) from $I_{\text{eq}}$ resulting from noise increases as T. This supports the following

**Observation 4** All else equal, indicators with larger relaxation times T tend to have wider natural ranges of variation.

For typical forms of the noise, the distribution of I(t) in the absence of pressures follows a normal distribution with mean $I_{0}$. If σ is the standard deviation of this distribution, the natural range according to the definition above is given by $I_{\text{low}}=I_{0}-1.96\sigma$ and $I_{\text{high}}=I_{0}+1.96\sigma$.

The problem of computing the mean time to recovery is mathematically a problem of computing the mean first passage time of a univariate random process. In the special case that “noise” in the model above is white noise, the mean first passage time for reaching $I_{\text{low}}$ from a starting value $I_{1}<I_{\text{low}}$ for P=0 is (Gardiner, 1990)

(4) $$\sqrt{\frac{\pi }{2}}T\overset{-1.96}{\underset{\left(I_{1}-I_{0}\right)/\sigma }{\int }}\exp\left(\frac{y^{2}}{2}\right)\left[1-\text{erf}\left(\frac{y}{\sqrt{2}}\right)\right]\text{d}y\text{,}$$

with erf(x) denoting the so-called error function. The lower bound of the indicator target range is the value of $I_{1}$for which the expression above equals R. Fig. 2 illustrate the resulting dependence of $I_{1}$ on T.

As can be seen in Fig. 2, the target range quickly becomes very wide when T is less then about half as large as R, and differs only little from the natural range for T>10R. The actual value of T therefore typically matters only when it is within about 0.5R to 10R.

For relaxation times T much smaller than the maximal mean recovery time R, corresponding to $\left(I_{1}-I_{0}\right)$ much larger than σ, noise can be disregarded and the pressure-free relaxation of I(t) approximated by a simple, exponential relaxation. For the case that $I\left(t\right)=I_{1}$ at t=0, one gets $I\left(t\right)-I_{0}=\left(I_{1}-I_{0}\right)\exp\left(-t/T\right)$. Interpreting $I_{1}$ as the lower bound of the target range, the condition $I\left(R\right)=I_{\text{low}}$ then leads to $I_{1}=I_{0}-1.96\sigma \exp\left(R/T\right)$. Correspondingly, the condition for sustainable use becomes $I_{0}-1.96\sigma \exp\left(R/T\right)<I\left(t\right)<I_{0}+1.96\sigma \exp\left(R/T\right)$.

## Figures and Tables

**Table 1:** Comparison of concepts of weakly and strongly sustainable use.

	Weakly sustainable use	Strongly sustainable use
Types of relevant services	Societal choice	A priori unknown
Value of services used	Mostly known	Unknown or uncertain
Value to be preserved	Anthropogenic capital plus natural capital	Natural capital
Nature of typical target	The point corresponding to optimal long-term use	The range allowing timely recovery
Management philosophy	Optimal control (as in control theory)	Limitation of pressures

## Footnotes

1 Others motivate strong sustainability by non-substitutability of critical natural capital, incomprehension of natural systems, irreversibility of losses, and ethically (Dietz and Neumayer, 2007).

2 A 5% probability of failing to meet the target for the state indicator must be admitted for consistency with the 5% probability that state indicators fall outside the natural range even in absence of pressures. To see this, consider state indicators with recovery rates much slower than 1/R, for which the target range becomes essentially identical to the natural range (Appendix, Fig. 2).

3 The first condition is likely to be stronger for state changes involving extinctions or ecosystem bi-stability, the second condition in situations where mean recovery time is a smooth function of the indicator value.

4 The “noise” term is assumed to have a long-term mean of zero. If not, this can be enforced by adjusting the value of $I_{0}$.

5 This assumes autocorrelation time is not much larger than T.
